# Fano resonances from gradient-index metamaterials

**DOI:** 10.1038/srep19927

**Published:** 2016-01-27

**Authors:** Yadong Xu, Sucheng Li, Bo Hou, Huanyang Chen

**Affiliations:** 1 College of Physics, Optoelectronics and Energy & Collaborative Innovation Center of Suzhou Nano Science and Technology, Soochow University, No. 1 Shizi Street, Suzhou 215006, China

## Abstract

Fano resonances – resonant scattering features with a characteristic asymmetric profile – have generated much interest, due to their extensive and valuable applications in chemical or biological sensors, new types of optical switches, lasers and nonlinear optics. They have been observed in a wide variety of resonant optical systems, including photonic crystals, metamaterials, metallic gratings and nanostructures. In this work, a waveguide structure is designed by employing gradient-index metamaterials, supporting strong Fano resonances with extremely sharp spectra. As the changes in the transmission spectrum originate from the interaction of guided modes from different channels, instead of resonance structures or metamolecules, the Fano resonances can be observed for both transverse electric and transverse magnetic polarizations. These findings are verified by fine agreement with analytical calculations and experimental results at microwave, as well as simulated results at near infrared frequencies.

In the past few years, the development of artificial metamaterials provides us with feasible techniques to manipulate the propagation of electromagnetic (EM) wave or light^1^,^2^. Using metamaterials, many fascinating physical phenomena have been demonstrated, including negative refraction^3^,^4^,^5^, invisibility cloaks^6^,^7^,^8^ and other optical devices based on transformation optics^9^,^10^. Metasurfaces – a class of ultrathin and planar metamaterials – extend these capabilities even further. The interaction between light and metasurfaces has given rise to numerous potential applications^11^,^12^,^13^,^14^,^15^,^16^,^17^,^18^,^19^,^20^,^21^,^22^,^23^,^24^,^25^,^26^,^27^,^28^, ranging from anomalous reflection or refraction of light^11^,^12^ and ultrathin flat lenses^15^ to photonic spin Hall effect^19^ and manifestation of PT symmetry breaking in polarization space^24^. Utilizing the concept of metasurfaces, gradient-index metamaterials (GIMs) have drawn much attention. It was shown previously that the GIMs can be employed to convert an incident propagating wave (PW) into a surface-like wave (SW) with high efficiency^27^. By integrating two identical GIMs into a paralleled-plated waveguide, broadband asymmetric propagation of light independent of its polarization^28^ was reported. In that case, the GIMs enable mode conversion, i.e. converting the guided mode gradually into a SW mode without any scattering, or vice versa.

Fano resonances, which exhibit an asymmetric spectral profile, have also become a hot research topic thanks to their range of potential applications^29^,^30^,^31^. They are caused by the constructive and destructive coherent interference of a narrow discrete resonance with a continuous spectrum. As a general wave phenomenon, Fano resonance can be observed in a wide variety of resonant optical systems, including photonic crystals^31^, metamaterials^32^, plasmonic nanostructures^30^,^33^, semiconductor nanostructures^34^, and even silicon metasurfaces^35^,^36^. Here we design a symmetric waveguide structure by employing GIMs, within which we see a pronounced Fano resonance. The underlying mechanism involves the coherent interference of guided modes coming from different channels. Owing to the extremely sharp spectra and polarization-independence of this resonant phenomenon, our work opens up opportunities for designing optical sources such as terahertz lasers^37^.

## Results and Discussion

### Theoretical analysis

**Figure 1:**
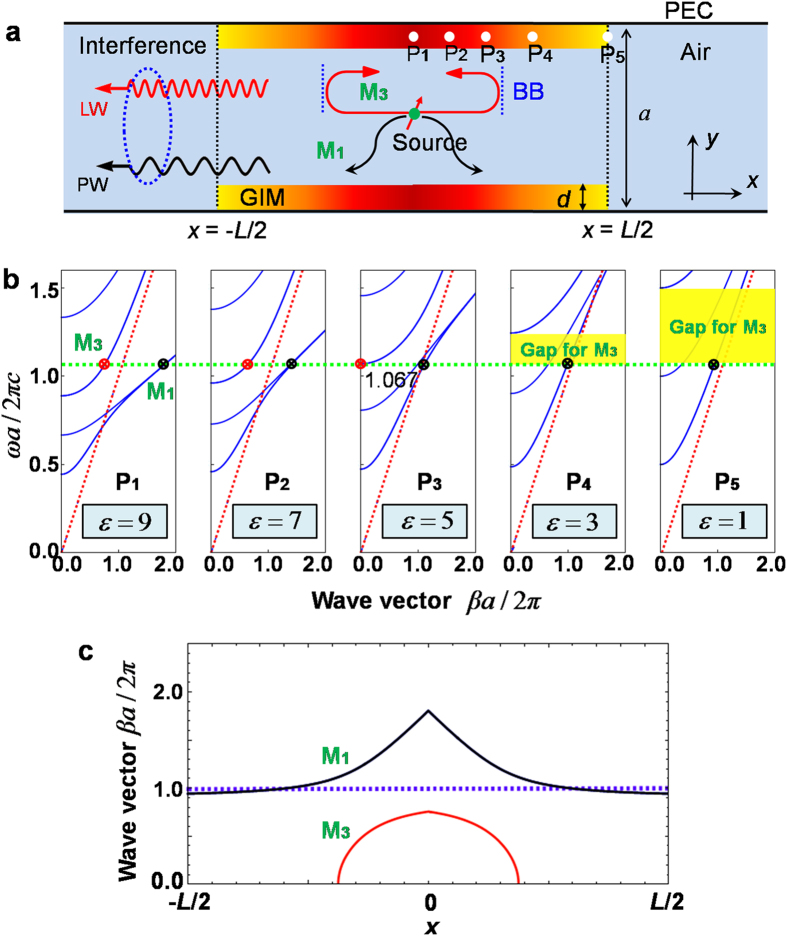
Concept of the designed waveguide structure exhibiting Fano resonances. (**a)** Schematic of a paralleled-plated waveguide made from two identical GIMs. The waveguide structure is bounded by walls of perfect electric conductors (PECs). Two black dashed lines illustrate the studied region, in which an excited source, shown by a red arrow with a green point, is located at the centre with coordinate (0,0). Two blue dashed lines show the band bottom (BB) of mode M_3_ at 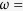
1.067. (**b**) The five discrete dispersion relations for a TE wave, corresponding to five different positions of GIMs (depicted by five white points in Fig. 1a). From left to right, the permittivity is 9, 7, 5, 3 and 1, respectively. The green dashed line shows a working frequency at 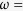
1.067, in which two guided modes M_1_ and M_3_ are indicated by black and red point, respectively. The red dashed lines represent light lines. **c**, The wave vectors of modes M_1_ and M_3_ at different positions *x* in the above structure, at working frequency 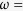
1.067.

The Fano effect is illustrated in Fig. 1. A symmetrical waveguide structure, consisting of a two-dimensional paralleled-plated waveguide and two identical layers of GIMs (graduated regions in Fig. 1a), is designed to support the constructive and destructive interference of a narrow resonance with a continuous spectral line. The width of the waveguide is *a*. We assume that the filled medium is air. For GIMs of thickness *d* and length *L*, the index profiles are expressed as^27^,^28^


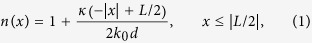


where 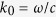 is the wave vector in free space, 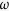 is the angular frequency of the EM wave, *c* is the velocity of light, and 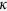 is a momentum parameter for designing GIMs^27^,^28^. We consider nonmagnetic GIMs, such that 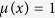. The permittivity profiles of the GIMs are thus 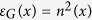.

The configuration shown in Fig. 1a is a symmetrical structure, in contrast to the previous work^28^, where because of the difficulty of analysing the band structures of the whole design, several dispersion relations at different positions were used to uncover the underlying physics, including the evolutions of guided modes inside the waveguide system. We use the same strategy for the current structure to explore Fano resonances within it. For a given set of parameters 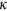, *L* and *d*, the index profiles are determined by equation 1. As an example, and without loss of generality, we set 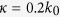, *L* = 40*d* and *d* = *a*/8. Then the permittivity of the GIMs changes from 1 to 9 as *x* varies from −*L*/2 to 0, and from 9 to 1 as *x* from 0 to *L*/2. Briefly, we first focus on the transverse electric (TE) polarization, that is, the electric field only along z direction. By applying the dispersion equations that we have obtained in ref. 28, Fig. 1b presents five dispersion relations corresponding to five positions (i.e., P_1_, P_2_, P_3_, P_4_ and P_5_ in Fig. 1a), where the permittivity 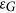 is 9, 7, 5, 3 and 1, respectively. In particular, the value of 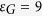 is related to the middle position, while the value of 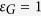 is located at external boundaries in the structure assumed here.

What will happen if an electric line current source is located in the centre, as shown by a red arrow with a green point in Fig. 1a ? To illustrate the underlying physics conveniently, the working frequency is chosen as 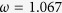, which coincides with the cutoff frequency of mode M_3_ at position P_3_ (see the third band in dispersion of 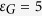 in Fig. 1b). Initially, the loaded source excites two symmetric modes, M_1_ and M_3_ (as shown by black and red points in the first plot of Fig. 1b). To quantify both modes propagating into the waveguide, their wave vectors 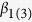 at different positions *x* were analyzed (Fig. 1c). Clearly, at the centre point 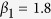 for mode M_1_ and 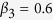 for mode M_3_. Afterwards, both guided modes will propagate simultaneously in the *x* and -*x* directions. As they propagate, the values of wave vectors decrease gradually due to the decreasing index of GIMs (Fig. 1c). When they arrive at positions 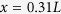 and 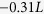 where the permittivity of GIMs is 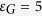, then 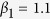 for M_1_ and 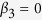 for M_3_. By checking the dispersion relations here (see the third plot in Fig. 1b), the mode M_3_ arrives at its band bottom. Subsequently the two modes propagate separately from each other. Mode M_1_ continues to propagate forward and exits from the structure freely, and its wave vector decreases continuously in this process (see Fig. 1c,b). Eventually, it propagates into the empty waveguide as a PW with 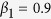. This PW from the M_1_ channel serves as the continuous radiation required by Fano resonances. For mode M_3_, it is not so straightforward. At its band bottom, two physical events take place simultaneously. One is the inter-mode transition from the third band to the first band^28^, which indicates that a little of the energy of mode M_3_ is transferred into mode M_1_, which subsequently leaks out of the designed structure into the empty waveguide with wave vector 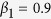. We regard this part of mode M_1_ sourced from M_3_ channel as a leaked wave (LW) (see Fig. S3 in ref. 28). The PW and LW in the empty waveguide will interfere with each other, but this interaction is not significant because the inter-mode transition is weak.

The other event is that the majority of mode M_3_ is reflected back from both directions *x* and −*x*, resulting from the band gap for M_3_ (see yellow regions marked on the last two plots in Fig. 1b). In fact, the two band bottoms of mode M_3_ form a Fabry-Pérot cavity, being analogous to the optical cavity made from a pair of highly reflective mirrors in a laser. Inside this cavity, the mode M_3_ oscillates back and forth along the *x* direction. Once the resonance conditions 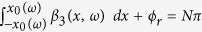 are satisfied (where 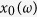 and 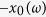 indicate the positions of band bottoms of mode M_3_ at working frequency 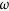, 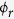 is the total reflected phase introduced by the band bottoms and *N* is a positive integer), the LW will grow more intense, leading to remarkable coherent interference in the empty waveguide. In this way, the Fabry-Pérot resonances provide the condition for narrow discrete resonances, as required for the Fano effect (see Fig. 1a). In addition, due to the multiple scattering or reflections, there are a series of Fano peaks and dips in transmission spectra.

**Figure 2:**
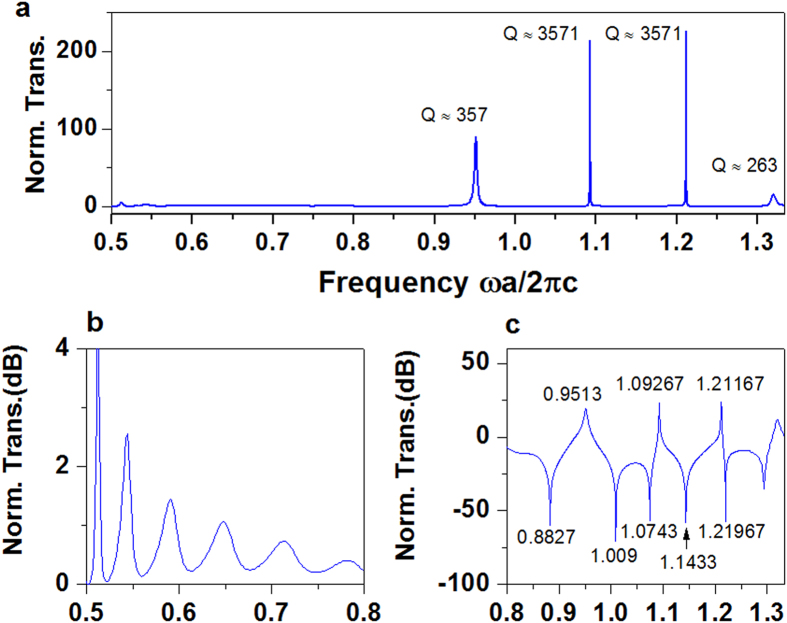
Calculated transmission, illustrating Fano resonances from the designed waveguide system. (**a**)Normalized transmission when the TE source is located at the centre of the structure. The peaks are located at frequencies of, from left to right, 0.95130, 1.09267, 1.21167 and 1.32000, respectively. (**b**,**c**) Normalized transmission of (**a)** divided into two sections and re-plotted in a dB scale. In (**c**) the deep positions (minima) are at 0.88270, 1.00900, 1.07430, 1.14330 and 1.21976, respectively.

To verify this intuitive interpretation, we carried out numerical calculations using a finite-element method; the normalized transmission of the TE source is shown in Fig. 2. The cut-off frequency of M_1_ in the empty waveguide is 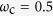, and at position *x* = 0 the cut-off frequencies of mode M_3_ and M_4_ are, respectively 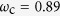 and 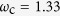 (first plot in Fig. 1b). For simplicity we focus on frequencies of 0.5 to 1.33, in which only modes M_1_ and M_3_ are excited by the centred source. In Fig. 2a there are four resonance peaks in the transmission spectrum, with quality factors *Q* up to 357, 3571, 3571 and 263, respectively. Here the factor *Q* is well extracted by fitting each resonance with theoretical formulas of typical Fano lineshape^35^. The details can be found in Supplementary Information (SI) (see Supplementary Note 1 and Supplementary Fig. 1). Comparing with previous works about Fano resoances at infrared frequencies^35^,^36^ or at terahertz region^38^, our proposed structure possesses very high Q factors. To inspect the transmission curve clearly, we re-plot it in two sections using a dB scale (Fig. 2b,c). For working frequencies of 0.5–0.8, the curve in Fig. 2b indicates the characteristic of Fabry-Pérot resonances, resulting from the scattering of mode M_1_. For frequencies 0.8–1.33 (Fig. 2c), besides the aforementioned peaks in Fig. 2a a series of sharp resonance dips emerge. By examining all peaks associated with resonance dips at frequencies between 0.89 and 1.33, we can see the typical features of Fano resonances: a sharp dip in amplitude followed by a sharp peak, or vice versa. These strong Fano resonances do not come from the scattering of mode M_1_ itself, but from the instructive or destructive coherent interference between the PW from M_1_ and LW from M_3_.

**Figure 3:**
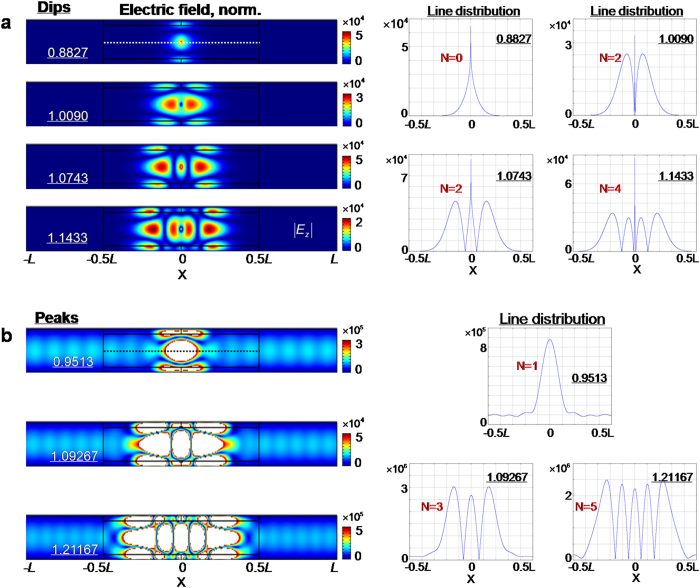
Electric field distributions at resonance dips and peaks. (**a**) Resonance dips. The left panel shows the field patterns of electric field density in the waveguide system, at frequencies 0.8827, 1.0090, 1.0743 and 1.1433, respectively. The right panel shows the line distributions of electric field density along the middle line (shown by the dashed white line in left panel), at the four frequencies. (**b**) Resonance peaks. As in (**a**) the left panel shows the field patterns, while the right presents the corresponding line distributions.

To inspect the discrete Fabry-Pérot resonances of mode M_3_, it is quite straightforward for us to examine the related field distributions, especially at the resonance dips and peaks. For dips, as shown by the left panel in Fig. 3a, the simulated electric-field distributions show clear and strong standing-wave patterns along *x* axis, while almost all the EM energy is confined inside the waveguide structure, with only a tiny field in the empty part of the structure. The right part of Fig. 3a shows the corresponding line distributions along the middle segment from −0.5*L* to 0.5*L* (dashed white line). In each plot, we observe the even-numbered anti-nodes in electric field density, if we ignore the singularity due to the source at the centre. For resonance peaks, similar results are found (Fig. 3b), including field patterns (left panel) that show standing waves, and line distributions (right panel) along the same segment. Inside the waveguide structure, the EM fields are greatly enhanced, and the EM wave spreading into the empty waveguide is dominated by mode M_1_, with an amplitude much larger than that in the absence of GIMs. For comparison, in the SI, we explore an empty waveguide containing two parallel dielectric blocks of high index, similar to the optical cavity of a laser. When a line source is centered, the transmission spectra feature a series of symmetric peaks (see Supplementary Fig. 2).

**Figure 4:**
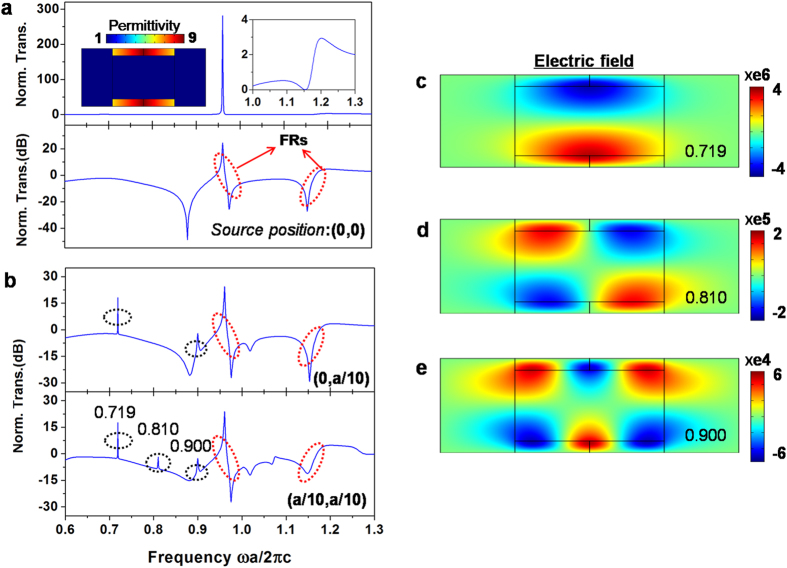
The robust features of the designed structure for achieving Fano resonance. (**a)** Calculated transmission when a TE source is located at centre (at position (0,0)) in the waveguide with GIMs whose refractive index ranges from 2.5 to 3. The upper panel shows the normalized transmission, and the inset shows the structure under consideration as well as the index distribution. The lower panel presents the same results in dB form, illustrating clearly the characteristic of all resonances in the spectrum. Two dashed red circles indicate the two Fano resonances that emerge. (**b**) Calculated transmission when the TE source deviates from the centre. The upper panel is for a source at position (0, *a*/10), while the lower panel is for a source at position (*a*/10, *a*/10). The dashed black circles indicate several peaks in the spectrum, originating from the resonances of mode M_2_. (**c**–**e**) Electric field distributions corresponding to resonance frequencies at 0.719, 0.810 and 0.900, respectively. Here the width of GIMs and the length of the whole structure are *d* = *a*/10 and *L* = 1.25*a*.

The structure we have considered here is just a special case for achieving strong Fano resonances. The index profiles of GIMs could be very robust. The only requirement for achieving these resonant features is that the structure be a symmetrical arrangement of two slabs of GIMs associated with the metallic waveguide, and that each GIM has a symmetrical index profile with a refractive index that changes gradually (and over a sufficient range) along the *x* direction. With this satisfied, the dispersion relations of guided modes at each position are modified gradually, and a cavity for a higher mode appears within some frequencies range. Such a requirement is so easy to meet that we can design a simpler and more robust version. For instance, by shortening the GIMs in Fig. 2, we studied a case where the index of all GIMs is changed from 1 to 1.4, and the length of the whole structure is 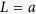. Supplementary Fig. 3 shows the calculated transmission of a line source at the centre, from which an obvious Fano effect can be seen at a higher frequency. On the other hand, it is not necessary for the starting index of GIMs to coincide with the background medium of the waveguide, that is 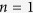. As a simple demonstration, we examined another structure with four GIMs whose index changes from 2.5 to 3. The transmission of a TE source at the centre is shown in Fig. 4a; two Fano resonances can be clearly observed at frequencies of around 0.96 and 1.15.

We also examine the situation when the line source is not located at the centre but is shifted slightly to one side. In such a case, because the symmetry of the waveguide system with respect to the added source is broken, not only the modes M_1_ and M_3_, but also an asymmetric mode M_2_ can be excited by added a source simultaneously at some frequency. When mode M_2_ escapes from the structure, owing to the mode-orthogonality, there is no interference between modes M_1_ and M_2_. As a result, the contribution of mode M_2_ is simply added linearly to the spectra from all the relevant scattering channels. To further inspect the transmission characteristic of the inner source, for simplicity we consider the version in Fig. 4a. Figure 4b shows the corresponding transmission spectra for two different positions: one at (0, a/10), the other at (a/10, a/10). Fano resonances, stemming from the continuous radiation of mode M_1_ and the narrow resonances of mode M_3_ still remain, without any influence from the M_2_ contribution except that the resonance dips are a little bit higher (see the Fano resonances at frequency 1.15 in Fig. 4a,b). On the other hand, we can observe a series of distinct peaks in the spectra, denoted by the dashed black circles in Fig. 4b, which are the result of scattering of the mode M_2_ resonances by the GIMs. This is verified further by the electric field distributions at three frequencies 0.710, 0.800 and 0.900 (Fig. 4c–e).

### Proof of principle by microwave experiments

In principle, the proposed device works at frequencies from microwave to terahertz, and even in the near-infrared region, as long as the GIMs with the required index profile can be fabricated. At a higher frequency, the assumption of perfect electric conductors of the waveguide is invalid: they should be replaced by two blocks of real metals, for example silver. In SI, we report on a concrete example where the separation of the two blocks of silver is *a* = 1600 nm, and the index profiles of GIMs still range from 2.5 to 3 in a length of 1000 nm. Among wavelengths from 1000 nm to 2000 nm, two obvious Fano resonances can be observed at the wavelengths of around 1221 nm and 1745 nm (see Supplementary Fig. 4). Because the interaction of silver and TE wave is not strong and most of EM energy is confined in both regions of GIMs and core medium, the influence of metal loss on Fano resonances is insignificant, leading to a high *Q* (e.g., the resonance around 1745 nm).

**Figure 5:**
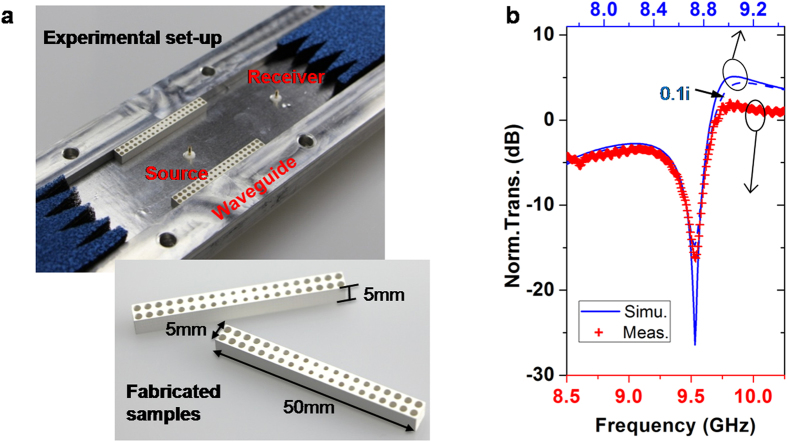
Experimental verification of Fano resonances. (**a**) The experimental set-up and fabricated samples. The width and height of the waveguide are *a* = 40 mm and *h* = 5 mm, respectively, and the length, width and height of samples are *L* = 50 mm, *d* = 5 mm and *h* = 5 mm, respectively. The source is positioned at the centre, and the receiver is located out of the designed region, 25 mm from its outer boundary. (**b**) The normalized transmissions of experimental measure (red crosses) and simulated analysis (blue curves). In the simulations, the two slabs of GIMs are replaced by designed samples, that is, by two dielectric substrates with different holes. The blue solid curve is for substrate without loss, while the blue dashed one is for substrate of 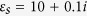.

To test our idea, we performed microwave experiments. The implementation was based on the simplified version in Fig. 4; Fig. 5a shows the experimental setup, consisting of a waveguide with two GIMs. The parameters are *a* = 40 mm, *d* = 5 mm and *L* = 50 mm. Index profile ranging from 2.5 to 3 was obtained by drilling air holes with different sizes in a dielectric substrate with 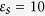 (Supplementary Table 1 shows the sizes in detail). Figure 5b shows a Fano resonance measured at around 9.5 GHz; the simulated results of the same structure are shown for comparison. They are almost consistent, except for a blueshift of the experimental resonance position of 0.8 GHz. This shift is caused mainly by the defects in the sample – Fano resonances are usually very sensitive.

In the same configuration, similar physics can be uncovered when the TE polarization is changed to TM (transverse magnetic). For instance, the corresponding dispersion relations of guided modes at each position are modified by GIMs, the effect of the inter-mode transition (from M_2_ to M_0_) happens at the band bottom of mode M_2_, and the Fabry-Pérot cavity for mode M_2_ can be found at some frequencies. Consequently, when a TM source is centred, the transmission spectra show a number of strong Fano resonances originating from constructive and destructive coherent interference between the continuous spectrum of mode M_0_ and narrow Fabry-Pérot resonances of mode M_2_ confined in GIMs (see Supplementary Fig. 5 and Supplementary Note 2).

## Conclusions

We have analyzed theoretically strong Fano resonances from a simple and feasible waveguide containing GIMs, and have verified their existence experimentally in microwave experiments. The underlying mechanism is the interaction of guided modes from different channels. The fact that the proposed system works independently of light polarization allows us to extend it to three-dimensional cases, e.g., a cylindrical waveguide with GIMs. We expect that such Fano resonances should also be experimentally accessible at terahertz and even infrared frequencies. The size of our proposed structure is moderate. For instance, the length of the robust version in Fig. 4 is 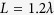, compared with the first resonance at 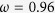. With optimization we can design a much simpler and smaller platform, which might find potential uses in sub-wavelength optics, sensing, optical sources, and on-chip applications.

## Methods

### Theory and simulations

The dispersion relations in Fig. 1b and the wave vectors in Fig. 1c were analyzed using the equations reported in ref. 28. The numerical calculations in Figs 2,4 and 5, including transmission and field patterns, were carried out by using the finite-element software COMSOL Multiphysics. The scattering boundaries were set for both sides of the waveguide, and the line source of TE polarization with a current 1 A is embedded inside the studied structures. For normalized transmission, when the GIMs were put inside waveguide, a transmission *T*_1_ of the source was obtained by integrating the Poynting vector along the cross-section near the output port. By removing all GIMs, another transmission *T*_2_ at the same position was found by using the same calculated method. Finally, the normalized transmissions were computed as *T*_1_/*T*_2_.

### Sample fabrications

In the experiments, two GIMs of size 50 mm × 5 mm × 5 mm (length, width and height), were fabricated by drilling different-sized holes in a dielectric plate with permittivity 10. The tables in SI shows the detailed diameters of all holes. Each sample consists of forty unit cells, and the size of unit cell is 2.5 mm × 2.5 mm × 5 mm.

### Experiments and measurements

The waveguide system is a home-made rectangular one with cross-sectional size of 40 mm × 5 mm. Two identical waveguide-to-coaxial cables were fixed in the middle line apart from the outer boundaries of the waveguide. One cable serves as the source, radiating a TE10 mode wave; the other acts as a probing cable that receives the electric field. The microwave network analyzer (Agilent N5230C), connecting to two cables, was used to provide the power source and record the transmission.

## Additional Information

**How to cite this article**: Xu, Y. *et al.* Fano resonances from gradient-index metamaterials. *Sci. Rep.* **6**, 19927; doi: 10.1038/srep19927 (2016).

## Supplementary Material

Supplementary Information
