# Supplementary material for: Fano resonances from gradient-index metamaterials
Source: Sci Rep. 2016 Jan 27;6:19927. doi: 10.1038/srep19927 (PMC4728681; doi:10.1038/srep19927)
Supplement: Supplementary Information [file srep19927-s1.doc]

**Supplementary Informations:**

**Fano resonances from gradient-index metamaterials**

Yadong Xu , Sucheng Li, Bo Hou, and Huanyang Chen

College of Physics, Optoelectronics and Energy & Collaborative Innovation Center of Suzhou Nano Science and Technology, Soochow University, No.1 Shizi Street, Suzhou 215006, China

***Supplementary Figures***

**
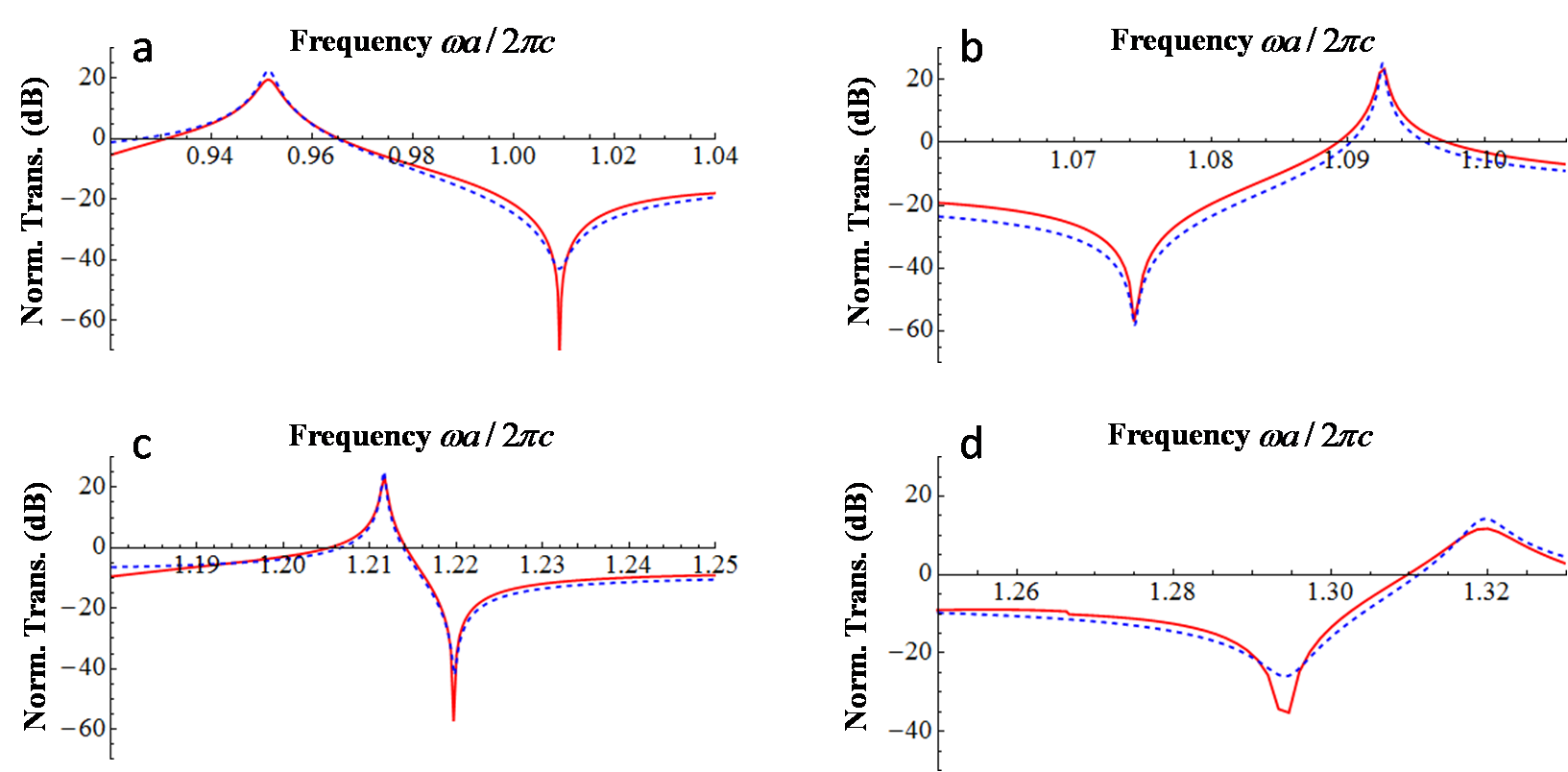
**

**Supplementary Figure 1 |** **Extracting the quality factors *Q* of Fano resonances in the transmission spectrum. a** is Fano resonance with peak at frequency $\omega=0.95130$. The red curve is numerical normalized transmission (in dB), while the blue dashed curve is the fitting result based on theoretical formula with parameters $\omega_{0}=0.95130$,$a_{1}=-0.31$, $a_{2}=0.0187\omega_{0}$ and $\gamma=0.0014\omega_{0}$. **b** is Fano resonance with peak at frequency $\omega=1.09267$. The fitting parameters are $\omega_{0}=1.09267$,$a_{1}=0.15$, $a_{2}=0.0025\omega_{0}$ and $\gamma=0.00014\omega_{0}$. **c** is Fano resonance with peak at frequency $\omega=1.21167$. The fitting parameters are $\omega_{0}=1.21167$,$a_{1}=-0.38$, $a_{2}=0.0026\omega_{0}$ and $\gamma=0.00014\omega_{0}$. **d** is Fano resonance with peak at frequency $\omega=1.32000$. The fitting parameters are $\omega_{0}=1.32000$,$a_{1}=0.51$, $a_{2}=0.0097\omega_{0}$ and $\gamma=0.0019\omega_{0}$.


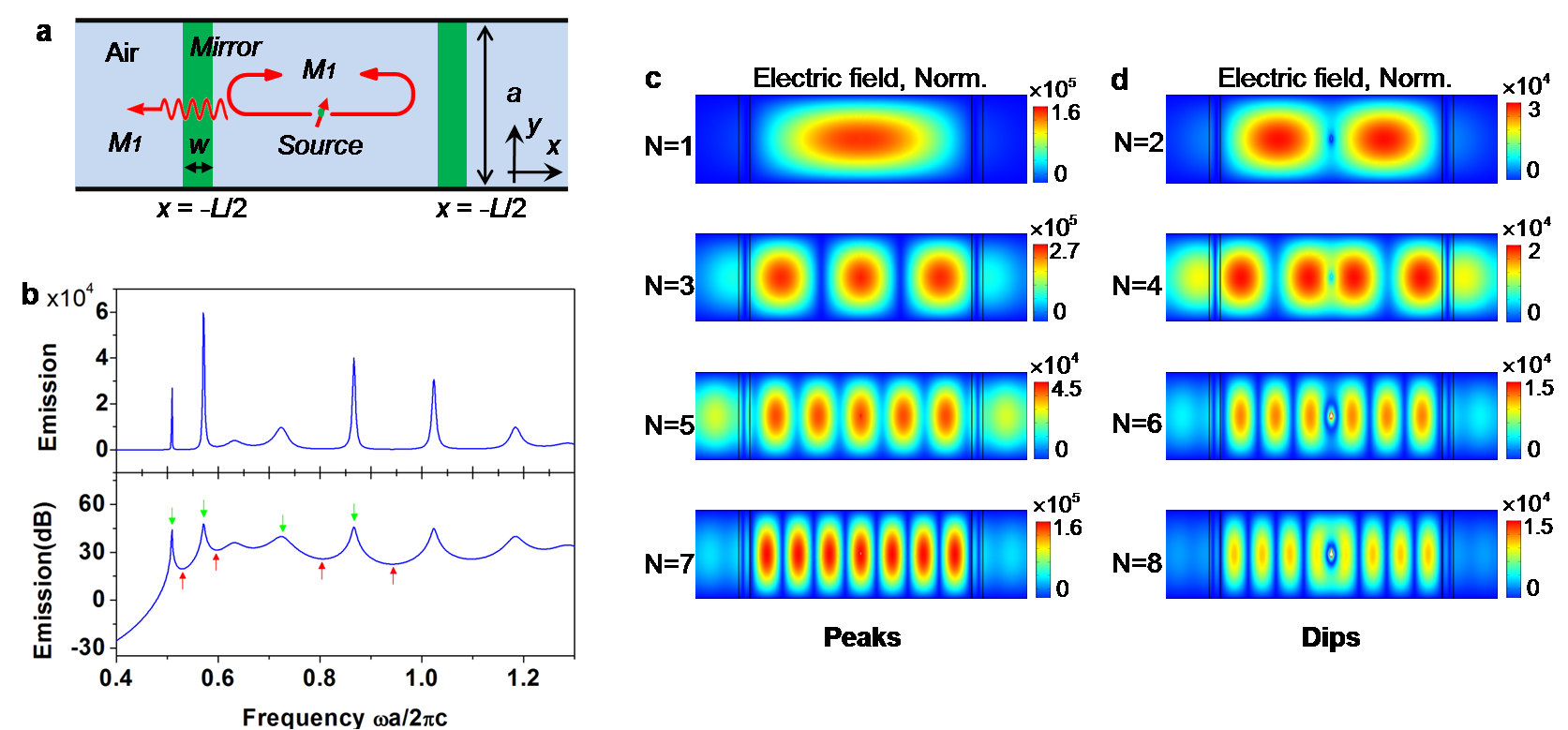


**Supplementary Figure 2** | **The physics of an optical cavity. a**, The schematic diagram of an optical cavity, a waveguide with two mirrors separated by air. We use two dielectric blocks of a width *w*=0.25*a* in numerical calculations to play roles of the two mirrors with high reflection. Their permittivity and permeability are set as $\varepsilon_{d}=10$ and $\mu=1$, respectively. **b**, The radiation emission of a TE line source located at the center of the optical cavity, which is numerically calculated by integrating the Poynting vector along the cross section near the output port. In the spectra, we observe a series of symmetric resonance peaks resulted from Fabry-Pérot resonances of mode M_1_ confined in the designed cavity. **c**, The field distributions of electric field density at several resonance peaks. From top to bottom, they correspond to four frequencies which are indicated by the green arrows in (**b**). **d**, The field distributions of electric field density at several resonance dips. From top to bottom, they correspond to four frequencies which are indicated by the red arrows in (**b**).


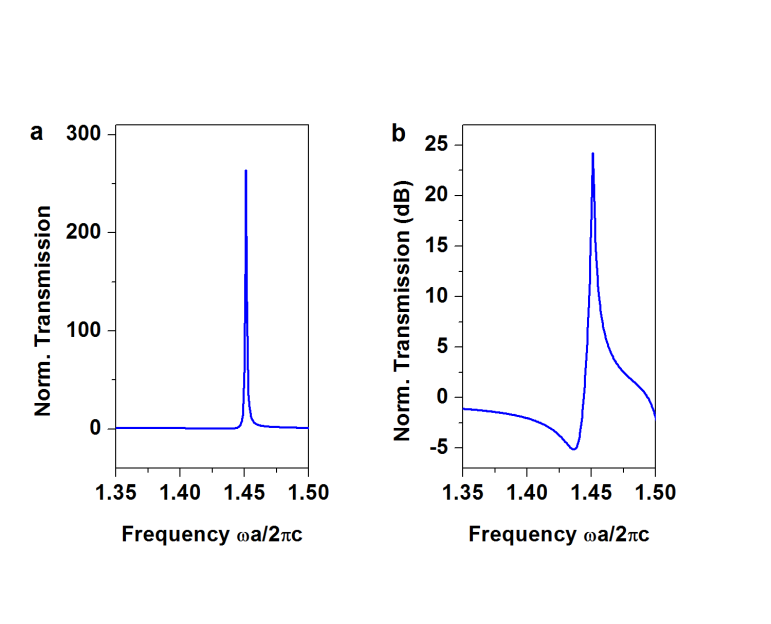


**Supplementary Figure 3** | **The robust feature verified by another designed structure where the index profiles of both gradient index meta-materials (GIMs) range from 1 to 1.4. a**, Normalized transmission of a TE line source at centre, where a strong Fano resonance appears at the frequency of $\omega=1.45$. **b**, The same as (**a**), but in a dB scale. Here the length of the whole structure is *L*=*a*.


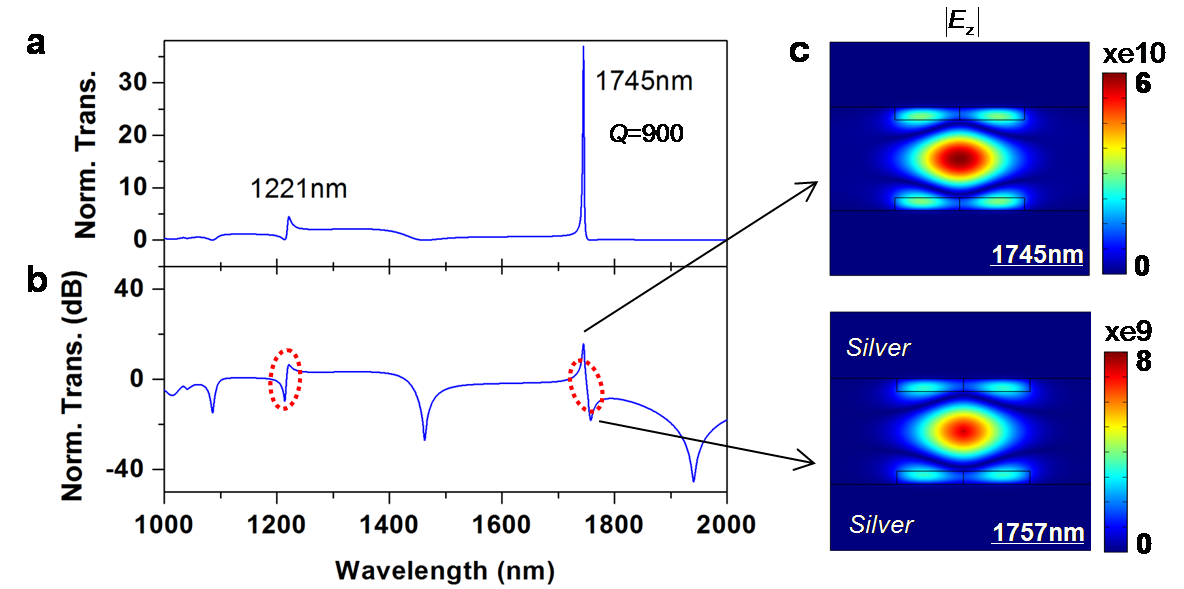


**Supplementary Figure 4** | **Fano resonances at near-infrared frequencies. a**, Normalized transmission for a TE line source at the centre. **b**, The same as (**a**), but in a dB scale. From the transmission spectra, two apparent Fano resonances can be observed. One is at the wavelength around 1221nm, and the other is at 1745nm, with a quality factor Q of about 900. **c**, The distributions of electric field density at 1745nm and 1757nm. In calculations, the separation of the two blocks of silver is *a*=1600nm. For GIMs, it has *L*=2000nm and *d*=200nm, and their index profiles are identical to those in Fig. 4, ranging from 2.5 to 3. For real metals (Supplementary Ref. 1), silver, its dielectric function is given by $\varepsilon(\omega)=5-\omega_{p}^{2}/(\omega^{2}+i\omega\tau)$, where $\omega_{p}=14\times10^{15}s^{-1}$ and $\tau=0.032\times10^{15}s^{-1}$. Such an analytical expression agrees well with widely accepted experimental data from Supplementary Ref. 2, particularly at optical and near-infrared frequencies.


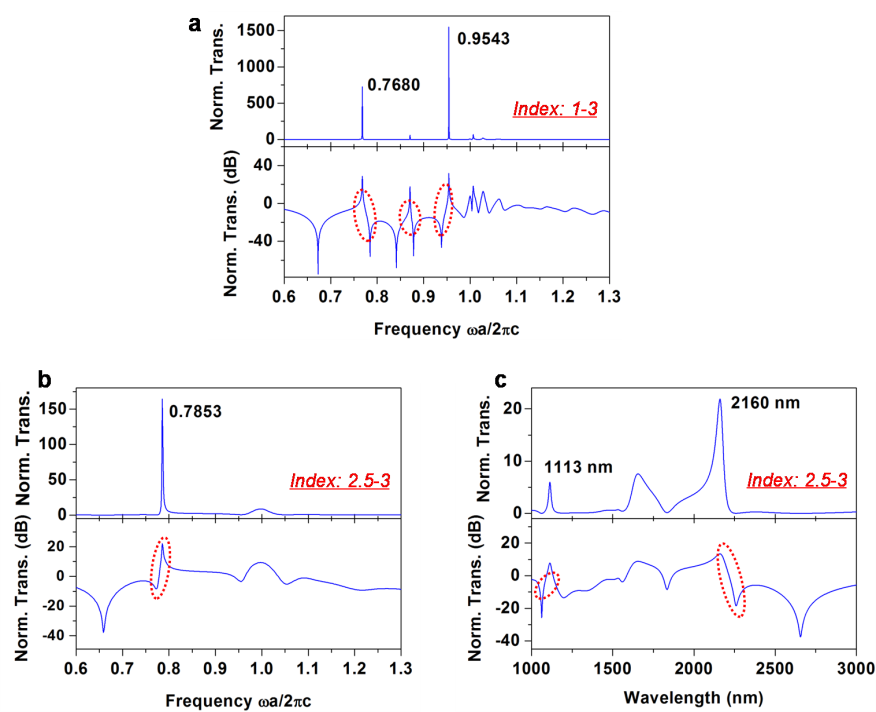


**Supplementary Figure 5** | **Theoretical analysis to demonstrate that the proposed structure works for TM polarizations. a**, Normalized transmission when the TE source in the case of Fig. 2 is changed to a TM source. **b**, Normalized transmission when the TE source in case of Fig. 4 is changed to a TM source. **c**, Normalized transmission when the TE source in case of Supplementary Fig. S3 is changed to a TM source. At this time, the surface plasmons are excited at the interfaces between silvers and GIMs. As a result, their resonant amplitudes are more sensitive to metal loss, in contrast with those of TE polarizations. In all plots, several Fano resonances are marked by red dashed circles. In calculations, the TM source is simulated by a tiny circle with $H_{z}=1 (A/m)$.

***Supplementary Notes***

***Supplementary Note 1.*** In the main text, Fig. 2a shows the normalized transmission when the TE source is placed at the centre of the structure. There are four Fano resonance peaks located at frequencies of, 0.95130, 1.09267, 1.21167 and 1.32000, respectively. By fitting each resonance with the theoretical formulas of typical Fano lineshape^3^, given by $I\text{=}\left|a_{1}+a_{2}/(\omega-\omega_{0}+i\gamma)\right|^{2}$, the quality factor of each resonance can be calculated by $Q\text{=}\omega_{0}/2\gamma$.

***Supplementary Note 2.*** In the main text, we only focus on the TE polarizations. In fact, due to the intrinsic mechanism of Fano resonances behind the proposed structure, similar phenomena should be seen in the same configuration under TM polarizations. To certify this point, we revisit three cases mentioned in the main body, i.e., the case of Fig. 2 where the index is changed from 1 to 3, the case of Fig. 4 where the index is changed from 2.5 to 3, and the case of Supplementary Fig. S4 at near-infrared frequencies. When the TE source is replaced by a TM source with other conditions unchanged, Supplementary Fig. S4 shows the corresponding spectra of transmission in the above three cases, where Fano resonances are clearly demonstrated as well.

***Supplementary Tables***

| **Permittivity of substrate** |  |  |  |  | **10** |  |  |  |  |  |
| --- | --- | --- | --- | --- | --- | --- | --- | --- | --- | --- |
| **Required refractive index** | **2.5** | **2.55** | **2.60** | **2.65** | **2.70** | **2.75** | **2.80** | **2.85** | **2.90** | **2.95** |
| **Diameters of air holes(mm)** | **1.8** | **1.70** | **1.70** | **1.60** | **1.50** | **1.50** | **1.40** | **1.30** | **1.20** | **1.10** |
| **Realized refractive index** | **2.52** | **2.59** | **2.59** | **2.67** | **2.73** | **2.73** | **2.79** | **2.84** | **2.89** | **2.94** |

**Supplementary Table 1. The detailed sizes of air holes in the dielectric substrate.** In the experimental design, the inhomogeneous GIMs with indexes from 2.5 to 3 are equally divided into 10 segments, each with a length of 2.5mm. For each segment, its index profile is replaced by a constant value. The required refractive index in this table lists ten values of the ten segments, which were realized by drilling air holes of different sizes in the dielectric substrate. In fabrication, the size of each unit cell is 2.5mmx2.5mm, and the total length of the sample is 50mm (2.5mmx20).

**Supplementary Reference:**

1. Cai, W. & Shalaev, V. M. *Optical Metamaterials: Fundamentals and Applications*, Ch. 2, 21-23 (Springer, 2009).
2. Johnson, P. B. & Christy R. W. Optical-constants of noble-metals. Phys. Rev. B 6, 4370–4379 (1972).
3. Yang, Y., Kravchenko, I., Briggs, D. & Valentine, J. All-dielectric metasurface analogue of electromagnetically induced transparency. Nature Commun. 5, 5753(2014).
